# Predictive Criterion Validity of the Parsley Symptom Index Against the Patient-Reported Outcomes Measurement Information System-10 in a Chronic Disease Cohort: Retrospective Cohort Study

**DOI:** 10.2196/53316

**Published:** 2024-02-16

**Authors:** Hants Williams, Sarah Steinberg, Kendall Leon, Ryan Vingum, Mengyao Hu, Robin Berzin, Heather Hagg, Patrick Hanaway

**Affiliations:** 1 Applied Health Informatics School of Health Professions Stony Brook University Stony Brook, NY United States; 2 Parsley Health New York, NY United States; 3 Untold Content Cincinnati, OH United States; 4 Family to Family Weaverville, NC United States

**Keywords:** chronic disease, eHealth, ePROM, mHealth, Parsley Symptom Index, patient-reported outcome measure, PROM, PSI, telehealth, telemedicine, validation, web-based

## Abstract

**Background:**

Approximately 60% of US adults live with chronic disease, imposing a significant burden on patients and the health care system. With the rise of telehealth, patient-reported outcomes measures (PROMs) have emerged as pivotal tools for managing chronic disease. While numerous PROMs exist, few have been designed explicitly for telehealth settings. The Parsley Symptom Index (PSI) is an electronic patient-reported outcome measure (ePROM) developed specifically for telehealth environments.

**Objective:**

Our aim is to determine whether the PSI predicts changes in the established Patient-Reported Outcomes Measurement Information System-10 (PROMIS-10) Global Health, a 10-question short form.

**Methods:**

We conducted a retrospective cohort study using data from 367 unique patients, amassing 1170 observations between August 30, 2017, and January 30, 2023. Patients completed the PSI and the PROMIS-10 multiple times throughout the study period. Using univariate regression models, we assess the predictive criterion validity of the PSI against PROMIS-10 scores.

**Results:**

This study revealed significant relationships between the PSI and PROMIS-10 physical and mental health scores through comprehensive univariate analyses, thus establishing support for the criterion validity of the PSI. These analyses highlighted the PSI’s potential as an insightful tool for understanding and predicting both mental and physical health dimensions.

**Conclusions:**

Our findings emphasize the importance of the PSI in capturing the nuanced interactions between symptomatology and health outcomes. These insights reinforce the value of the PSI in clinical contexts and support its potential as a versatile tool in both research and practice.

## Introduction

Approximately 60% of US adults live with one or more chronic diseases [1]. Due to the growing population of adults aged 65 years or older and increased risk factors, chronic disease is expected to impact over 221 million people in the United States by 2050 [2]. Developing tools and strategies to promote health is increasingly important as a way to alleviate the enormous burden that chronic disease places on patients, providers, and health care systems [3]. Compared with people without chronic disease, people with chronic conditions have higher health care costs and require more time to manage their care than primary care providers have available [4]. Many new care models, such as the Chronic Care Model, have been implemented [5-7] to overcome these challenges; however, more work is needed to increase access to effective chronic disease care that reduces resource constraints and improves patients’ health.

Emerging telehealth tools, which have become increasingly popular and widespread since the COVID-19 pandemic [8], are proving capable of creating meaningful changes in chronic disease management [9]. Care provided through telehealth has been shown to alleviate many of the burdens of chronic diseases, such as lowering health care costs, reducing missed appointments, and increasing access to timely care [10]. Telehealth tools have also been shown to encourage collaborative disease management, incentivizing patients to participate in their care [11,12].

Patient-reported outcomes measures (PROMs) are patient-oriented, self-reporting tools that can be implemented in a range of settings to improve care processes and track outcomes [13]. PROMs have been found to help facilitate patient-clinician communication [14] and save valuable time and resources for both patients and providers [15], making them a crucial tool in chronic disease management.

Several powerful PROMS exist to capture patients’ perceptions of their health and well-being, such as the Patient Reported Outcomes Measurement Information System (PROMIS) [16,17], the 36-Item Short Form Health Survey [18,19], and the Medical Symptom Toxicity Questionnaire [20]. However, few validated PROMS were designed for telehealth settings first, as opposed to paper and pen PROMs retrofitted for a telehealth environment.

In response to a need for a validated, digital-first electronic patient-reported outcome measure (ePROM), Parsley Health—a subscription-based, holistic medical practice—designed the Parsley Symptom Index (PSI) [21]. To our knowledge, the PSI is the only multi-item ePROM designed for and within a telehealth environment with internal validity. The Medical Symptom Toxicity Questionnaire, a conceptually similar functional medicine tool created over 20 years ago, was initially paper-based and has yet to be validated.

In previous validation studies, the use of the PSI in clinical practice was found to be feasible and acceptable to patients and clinicians [21,22]. The PSI also demonstrated internal validity when compared with the single-rated health (SRH) item for adults with chronic disease in a telehealth setting [22]. While there was a moderate level of association and agreement between the PSI and the SRH and the 2 instruments had conceptual similarities, the PSI captured additional granular changes in symptoms with treatment over time compared to the SRH measure, which remained relatively static [22]. This difference was expected, as the PSI is a 45-item ePROM and the SRH is a single question with a 5-item Likert scale response. As such, there is a need to compare it to a PROM that is closer in design and concept to the PSI, as well as to externally validate the PSI to determine its generalizability for use with different patient populations with chronic disease [23].

The primary objective of this study was to assess the criterion validity of the PSI against a validated, conceptually aligned, multi-item PROM (ie, the Patient-Reported Outcomes Measurement Information System-10 [PROMIS-10] Global Health, a 10-question short form) [16,17]. We aimed to ascertain whether the PSI could predict alterations in the widely accepted PROMIS-10 tool.

## Methods

### Ethical Considerations

This study used patient-reported survey data that were recorded so that participants were unidentifiable to the researchers. The institutional review board at Stony Brook University considered this study exempt (IRB2020-00429) from the Code of Federal Regulations Title 45 requirements.

### Study Design

This retrospective cohort study took place at the “Family to Family” medical clinic in the Southeast region of the United States between August 30, 2017, and January 30, 2023, among a sample of 367 participants with a range of chronic diseases. Additionally, for the purpose of PSI to PROMIS T-score calibration, an independent data set consisting of 122,591 assessments from 29,353 customers of Parsley Health was used to establish the PSI T-score conversion table detailed in Multimedia Appendix 1.

### The PSI

The PSI is a 45-item ePROM, similar to a Review of Symptoms, focusing on bodily domains and the most commonly reported symptoms associated with chronic diseases for each domain. The PSI assesses a patient’s perception of symptom burden. The PSI was developed using the Federal Drug Agency’s guidance for PROM development [24]. Items are grouped into 9 systems and ranked on a scale from 0 (asymptomatic) to 10 (extremely symptomatic). A total score is calculated with the following 4 cutoff ranges: “well” (0-24), “symptomatic” (25-43), “very symptomatic” (44-71), and “sick” (≥71). The PSI has shown clinical validity for use in clinical practice [22].

### The PROMIS-10

The PROMIS-10 is a single, generalizable, and validated PROM that can be used for various diseases and conditions. It is a shortened version of PROMIS that was developed to minimize respondent burden. This version is a 10-item, patient-reported questionnaire that was created as a general health assessment tool. Nine out of 10 questions on the PROMIS-10 are answered using a 5-point Likert scale, with the tenth question answered using a numeric rating scale. Results can be tracked in three different ways: (1) answers to each of the 10 questions can be evaluated separately, (2) answers can be grouped together to provide a global summary score, or (3) answers can be split into 2 groups to provide a global physical health score and a global mental health score.

We compared the PSI to the PROMIS-10, as it is similar to other general health short-form surveys and is widely adopted due to its ease of use. The PROMIS-10 has been shown to be valid and reliable in clinical settings for patients from the general population [16] and those living with chronic diseases [25,26]. Similar to its more extensive counterpart, the PROMIS-10 has undergone rigorous testing and validation across diverse age groups, including younger and older adults [27,28], and has proven to be reliable across a variety of clinical populations [29-31].

### Study Setting and Population

Family to Family is a hybrid (remote and in-person) functional and holistic medicine clinic for adults and children located in the Asheville, North Carolina, metropolitan area. The average patient age was 53.7 years old, and patients predominantly identified as female (73%). While race data were not available, the 2 clinicians at this practice report that their patients are predominantly White.

### Procedure

Patients and their caregivers were prompted to complete both the PSI and the PROMIS-10 through a password-protected electronic medical record web-based portal before each clinical visit. The PSI was added as a PROM to complete along with the PROMIS-10 because the clinicians believed it provided different insight as a Review of Symptoms to capture a more comprehensive view of patients’ symptomatology and progress over time.

Patients were required to complete the PSI and PROMIS before their first clinical visit. If both ePROMs were not completed before a patient’s first clinical visit at Family to Family, the visit was postponed or rescheduled. For all subsequent visits, completing the ePROMs was optional but encouraged. Participants were not compensated for completing the ePROMs. When preparing for the patient’s visit, Family to Family clinicians could view responses to both ePROMs in a patient’s electronic health record and use these responses to guide a clinical encounter. Clinicians were able to ask targeted questions about a patient’s symptoms and identify triggers that might contribute to the symptoms.

### Data Analysis Software

The data analyses were conducted using Python (version 3.10; Python Software Foundation) [32].

#### Statistical Methods

We conducted an analysis to explore the relationship between the PSI and the PROMIS-10. Initially, the raw scores of the PSI and the PROMIS-10 were transformed into T-scores [33,34]. The approach for PSI T-score conversion is detailed in Multimedia Appendix 1. Following T-score conversions, the underlying distribution characteristics of PSI T-scores, PROMIS physical T-scores, and PROMIS mental T-scores were evaluated for normality and distribution. D’Agostino and Pearson normality test were applied to each set of scores to assess the normality. Measures of skewness and kurtosis were calculated for each set of scores to provide insights into the distribution’s symmetry. Histograms with overlaid box plots were created for each set of scores to visually inspect their distributions.

#### Univariate Regressions

We performed 2 univariate regression models to assess the predictive criterion validity of the PSI T-scores on the PROMIS physical and mental T-scores. Due to the observed nonnormal distribution of the PSI T-scores (*χ*^2^_1169_=183.324; *P*<.001), generalized linear models with a Gaussian family and identity link function were chosen as the appropriate modeling approach. This choice accommodates the nonnormal distribution of the PSI T-scores by allowing for a linear relationship between the predictors and response without assuming that the residuals are normally distributed. The flexibility in the Gaussian family made it suitable for modeling the specific distributional properties of the PSI T-scores.

Our first univariate model examined the relationship between PSI T-scores (an independent variable) and PROMIS mental T-scores (a dependent variable), aiming to understand how the PSI is predictive of mental health as quantified by the PROMIS scale. The second univariate model focused on the relationship between PSI T-scores (an independent variable) and PROMIS physical T-scores (a dependent variable), aiming to understand how the PSI is predictive of physical health as quantified by the PROMIS scale. This approach provides insights into the effects of PSI on mental and physical health that are robust to distributional assumptions. Coefficients, SEs, and significance levels were reported to highlight the specific relationships.

#### Tables

Pivot tables were used to summarize the mean (SD) of the PSI T-scores, PROMIS physical T-scores, and PROMIS mental T-scores. The data were stratified by time order, reflecting different periods of assessment. Multiple pivot tables were generated to encapsulate the mean (SD) for each measurement, organized by the time period.

## Results

### Overview

In our data set, we analyzed a total of 1170 observations from 367 unique patients recorded between August 30, 2017, and January 30, 2023, from Family to Family. On average, participants completed the PSI 3.2 times and the PROMIS 3.4 times during the study period. Adhering to the guidelines for good reporting practices, the CHERRIES (Checklist for Reporting Results of Internet E-Surveys) [35] is included in Multimedia Appendix 2. Detailed patient demographics and general descriptions of the sample are delineated in Table 1. On average, participants reported experiencing 8 distinct symptoms or conditions. Excluding nutrient deficiencies, the most commonly reported diseases and health problems, as classified by *International Statistical Classification of Diseases, Tenth Revision* (*ICD-10*) codes, were other fatigue (82/367, 22.3%), anxiety disorder (81/367, 22.1%), hypothyroidism (81/367, 22.1%), and chronic fatigue (65/367, 17.7%), as outlined in Table 2.

The mean value for the PSI T-score is 40.808 (SD 7.00), with a minimum and maximum range of values between 25 and 56, indicating a broad spectrum of reported symptom states within the sample. Since the expected average for a typical population is 50, this lower mean suggests that the sample population exhibits a higher level of symptoms or less optimal health than the general population. The mean value for the PROMIS physical score is 47.952 (SD 8.114), which is slightly below the expected average of 50. This result also implies that the physical health of the sample population is somewhat below average. The minimum and maximum values for the physical T-scores range from 19.9 to 67.7, indicating a broad spectrum of physical health states within the sample. The mean value for the PROMIS mental score is 46.638 (SD 8.551), which is also below the expected average of 50. This suggests that the mental health of the sample population is also somewhat lower compared to the general population. The PROMIS mental ranges from a minimum of 21.2 to a maximum of 67.6, further indicating variation in mental health states within the sample. Additional descriptives for PSI and PROMIS T-scores across time are provided in Table 3.

**Table 1:** Patient descriptives.

Variable		Frequency	Total, n	
Age (years), mean (SD)		53.7 (17.1)	367	
Annual income (US $), median (IQR)		70,344 (54,597-72,575)	360	
Annual income (US $), mean (SD)		98,849 (40,259)	361	
Number of symptoms and conditions, mean (SD)		8.1 (5.4)	367	
Number of PROMIS^a^ surveys completed, mean (SD)		3.4 (3.1)	367	
Number of PSI^b^ surveys completed, mean (SD)		3.2 (3.1)	367	
**Sex, n (%)**				367
	Male	99 (27)		
	Female	268 (73)		
**Insurance, n (%)**				332
	Yes	325 (97.9)		
	No	7 (2.1)		
**Relationship to the insured^c^, n (%)**				332
	Self	302 (91)		
	Spouse	26 (7.8)		
	Dependent	4 (1.2)		

^a^PROMIS: Patient-Reported Outcomes Measurement Information System.

^b^PSI: Parsley Symptom Index.

^c^Relationship to the insured refers to the participant’s status as the primary beneficiary of the insurance policy. “Self” indicates the participant holds the policy in their own name. “Spouse” denotes the participant is covered under a policy held by their married partner. “Dependent” means the participant is covered under a policy due to their status as a dependent, typically a family member without independent coverage.

**Table 2:** Frequency of reported symptoms and conditions.

	Name	ICD^a^ code type	Frequency, n (%)
1	Deficiency of multiple nutrient elements	E	139 (37.9)
2	Vitamin D deficiency, unspecified	E	93 (25.3)
3	Other fatigue	R	82 (22.3)
4	Anxiety disorder, unspecified	F	81 (22.1)
5	Hypothyroidism, unspecified	E	81 (22.1)
6	Chronic fatigue, unspecified	R	65 (17.7)
7	Essential fatty acid deficiency	E	64 (17.4)
8	Pure hypercholesterolemia, unspecified	E	62 (16.9)
9	Other abnormal glucose	R	52 (14.2)
10	Irritable bowel syndrome with diarrhea	K	51 (13.9)
11	Autoimmune thyroiditis	E	50 (13.6)
12	Mixed irritable bowel syndrome	K	50 (13.6)
13	Abnormal level of hormones in specimens from other organ or tissue	R	47 (12.8)
14	Other disorders involving the immune mechanism, Not elsewhere classified.	D	45 (12.3)
15	Gastroesophageal reflux disease without esophagitis	K	41 (11.2)
16	Essential (primary) hypertension	I	39 (10.6)
17	Major depressive disorder, recurrent, unspecified	F	39 (9.3)
18	Disorder involving the immune mechanism, unspecified	D	36 (9.8)
19	Irritable bowel syndrome with constipation	K	34 (9.3)
20	Impaired glucose tolerance (oral)	R	32 (8.7)

^a^ICD: International Classification of Diseases.

**Table 3:** Descriptive statistics by time order.

Time point	Sample size, n	PSI^a^ T-score, mean (SD)	PROMIS^b^ physical T-score, mean (SD)	PROMIS mental T-score, mean (SD)
1	362^c^	39.1 (6.7)	46.8 (8.3)	45.7 (8.6)
2	221	41.4 (7.0)	48.7 (8.1)	46.8 (8.7)
3	144	40.9 (7.2)	47.8 (8.4)	46.4 (8.5)
4	109	41.3 (6.7)	48.1 (8.2)	46.6 (9.0)
5	92	41.2 (6.7)	48.7 (7.2)	47.8 (8.3)
6	65	41.0 (6.5)	48.4 (6.7)	46.6 (8.0)
7	48	41.2 (6.0)	47.3 (7.5)	47.8 (8.1)
8	35	43.0 (7.4)	47.9 (8.7)	46.8 (7.5)
9	30	41.7 (7.2)	48.2 (8.5)	46.5 (7.8)
10	18	42.6 (7.1)	49.2 (7.7)	47.8 (8.1)
11	14	42.9 (7.9)	48.7 (7.8)	47.4 (9.3)
12	11	43.2 (9.5)	47.7 (6.5)	48.3 (9.4)
13	8	47.8 (8.4)	54.8 (7.1)	53.5 (9.3)
14	7	45.8 (6.9)	50.5 (11.8)	49.6 (6.2)
15	4	48.9 (6.0)	57.9 (3.2)	53.6 (6.9)
16	2	51.5 (3.5)	59.8 (3.0)	55.4 (10.0)

^a^PSI: Parsley Symptom Index.

^b^PROMIS: Patient-Reported Outcomes Measurement Information System.

^c^Of the 367 unique participants, 5 were excluded from time point 1 due to corrupted data files for the PSI or PROMIS assessments. This resulted in a sample size of 362 for the initial time point.

### Distributions And Normality

The distribution characteristics of the PSI and PROMIS T-scores were assessed for normality. The PSI T-scores were found to follow a non-normal distribution (*χ*^2^_1169_=183.324; *P*<.001). The skewness of 0.577 in the PSI T-scores indicates a distribution with a longer right tail and a concentration of scores on the left, reflecting a higher frequency of lower scores and thus a less healthy population. The negative kurtosis of –0.858 signifies a platykurtic kurtosis and that extreme outliers (very high or low) are less frequent in this data set than they would be in a normally distributed data set. In contrast, the PROMIS physical and mental T-scores were found to follow a normal distribution (*P*=.10 and *P*=.46), with a minor skewness of –0.145 and a kurtosis of 0.100 for the physical, while an almost perfect skewness of –0.015 and a slight platykurtic kurtosis of –0.173 for the mental.

### Univariate Regressions

#### Model 1: PSI T-Scores Predicting PROMIS Mental T-Scores

The first generalized linear regression model was fit using the Gaussian family with an identity link function, revealing a significant positive association between the PSI T-scores and PROMIS mental T-scores (Table 4). Specifically, a 1-unit increase in the PSI T-score corresponded to a 0.627-unit increase in the PROMIS mental T-score (95% CI 0.567-0.687; *z*=20.462; *P*<.001). The intercept was estimated at 21.0487 (95% CI 18.562-23.536). The model’s pseudo *R*^2^ value (CS) was 0.3008, indicating that it explained approximately 30.08% of the variability in the PROMIS mental T-scores. The deviance statistic, which measures the goodness of fit, was 62,925, and the Pearson chi-square value was approximately 62,900, further supporting the model’s fit to the data.

#### Model 2: PSI T-Scores Predicting PROMIS Physical T-Scores

The second generalized linear regression model was fit using the Gaussian family with an identity link function, revealing a significant positive association between the PSI T-scores and PROMIS physical T-scores (Table 4). Specifically, a 1-unit increase in the PSI T-score corresponded to a 0.6479 unit increase in the PROMIS physical T-score (95% CI, 0.593-0.703; *z*=23.064; *P*<.001). The intercept was estimated at 21.5112 (95% CI, 19.231-23.791). The model’s pseudo *R*^2^ value (CS) was 0.3653, indicating that it explained approximately 36.53% of the variability in the PROMIS physical T-scores. The deviance statistic, which measures the goodness of fit, was 52,883, and the Pearson chi-square value was approximately 52,900, further supporting the model’s fit to the data.

**Table 4:** Summary of generalized linear model regression models predicting Patient-Reported Outcomes Measurement Information System (PROMIS) mental and physical T-scores from Parsley Symptom Index (PSI) T-scores.

Model	Dependent variable	Coefficient	95% CI	*P* value	Pseudo *R*^2^
Model 1	PROMIS mental	0.6270	0.567-0.687	<.001	0.3008
Model 2	PROMIS physical	0.6479	0.593-0.703	<.001	0.3653

## Discussion

### Overview

Previous studies found the PSI to be a valid tool that can be deployed, completed, and helpful to both patients and clinicians [21,22,36]. This study examined differences in use between the PSI as compared to the PROMIS-10 short form when used in clinical settings with patients with chronic disease. The PSI and PROMIS-10 were chosen for this retrospective study because the clinicians believed it was useful for patients to complete both forms as they provide slightly different insights into how patients perceive their health status. While the differences between the PSI and PROMIS-10 reveal ways that each has its place in clinical practice, they overlap enough to demonstrate a moderate correlation to support validation of the PSI when compared to the PROMIS-10.

The overall mean (SD) statistics for the PSI and PROMIS-10 paint a picture of a sample population that is generally less healthy than the average population, both in terms of physical and mental well-being, as well as symptomatology. The lower PSI score, in particular, stands out, indicating a higher level of symptoms. These findings set the stage for further analysis to understand the underlying factors, relationships, and potential interventions that may be relevant to this specific population.

### Univariate Regression Analysis: Generalized Linear Model

The univariate generalized linear regression analyses conducted in this study used the Gaussian family with an identity link function to explore the relationship between the PSI T-scores and both mental and physical health as measured by the PROMIS scales. The consistency in the direction and significance of the relationships between the PSI across both mental and physical health domains defined by the PROMIS, as revealed in the univariate analyses, lends credibility to the models and provides a robust foundation for further exploration. The positive associations demonstrate the criterion validity of the PSI, illustrating its potential to predict changes in both mental and physical health as measured by the PROMIS scales. The substantial explanatory power of the models, as evidenced by the pseudo *R*^2^ values and supported by the deviance statistics and Pearson chi-square values, adds to the robustness of the findings and their potential implications for the PSI as a validated health assessment. This validation underscores the use of the PSI and its capability of offering insights into overall well-being. Future research may benefit from examining these relationships in different populations or contexts, potentially extending the applicability of the PSI.

### Limitations

This study bears several notable limitations. First, our data emanate from a single clinic where a significant majority of Family to Family participants identified as female (73%), with an average age of 53 years. Although race and ethnicity data were not available, the clinic reported that the patient population was predominantly White. Such skewness constrains the ecological validity of our findings.

Our sample size was not large enough to support a robust longitudinal analysis, thereby limiting the depth of insights we could derive. It is imperative for future validation studies to explore the PSI’s use within a more diverse demographic profile and over a more extended time frame. Such studies would not only deepen the understanding of symptom trajectories but also facilitate the evaluation of patient outcomes across a wider demographic landscape.

In terms of the PSI questionnaire itself, the Family to Family participants engaged with an earlier iteration. Based on patient feedback, Parsley Health implemented minor revisions to this version to enhance readability. Consequently, we made retrospective adjustments to ensure alignment with the updated version of the PSI, which encompassed an additional item but was more concise in terms of completion time since responses were no longer categorized as “resolved” or “ongoing.”

Additionally, the nature of this being a retrospective cohort study meant that the PSI and PROMIS-10 items were not presented to participants in a randomized manner, which could have potentially mitigated response biases. We advocate for the implementation of randomization, or A/B testing, in subsequent studies.

### Conclusions

Although we know that telehealth tools can be used to deliver effective care to patients with chronic conditions, few—if any—tools exist that are designed as digital-first ePROMS. This predictive criterion study compared the PSI—a digital-first ePROM—to the PROMIS-10, a traditional PROM, in a functional medicine clinic for patients with a range of chronic conditions. This study revealed significant relationships between the PSI and PROMIS physical and mental health scores through comprehensive univariate analyses, thus establishing support for the criterion validity of the PSI. These analyses highlighted the PSI’s potential as an insightful tool for understanding and predicting both mental and physical health dimensions.

Overall, the findings of this study emphasize the importance of the PSI as a versatile clinical instrument. Future research is warranted to further dissect these relationships and enhance our understanding of the PSI’s applicability in various health contexts.

## Appendix

Multimedia Appendix 1 Parsley Symptom Index (PSI) reverse coding guide.

Multimedia Appendix 2 CHERRIES (Checklist for Reporting Results of Internet E-Surveys) checklist.

## Abbreviations

CHERRIES: Checklist for Reporting Results of Internet E-Surveys
ePROM: electronic patient-reported outcome measure
ICD-10: International Statistical Classification of Diseases, Tenth Revision
PROM: patient-reported outcome measure
PROMIS: Patient-Reported Outcomes Measurement Information System
PSI: Parsley Symptom Index
SRH: single-rated health

## References

[1] (2022). Chronic diseases in America. Centers for Disease Control and Prevention.

[2] Ansah JP, Chiu CT (2023). Projecting the chronic disease burden among the adult population in the United States using a multi-state population model. Front Public Health.

[3] Østbye T, Yarnall KSH, Krause KM, Pollak KI, Gradison M, Michener JL (2005). Is there time for management of patients with chronic diseases in primary care?. Ann Fam Med.

[4] Wagner EH (1998). Chronic disease management: what will it take to improve care for chronic illness?. Eff Clin Pract.

[5] Lawn S, McMillan J, Pulvirenti M (2011). Chronic condition self-management: expectations of responsibility. Patient Educ Couns.

[6] Boehmer KR, Abu Dabrh AM, Gionfriddo MR, Erwin P, Montori VM (2018). Does the chronic care model meet the emerging needs of people living with multimorbidity? A systematic review and thematic synthesis. PLoS One.

[7] (2019). Fair Health study analyzes telehealth. FAIR Health.

[8] Corbett JA, Opladen JM, Bisognano JD (2020). Telemedicine can revolutionize the treatment of chronic disease. Int J Cardiol Hypertens.

[9] Guo Y, Albright D (2018). The effectiveness of telehealth on self-management for older adults with a chronic condition: a comprehensive narrative review of the literature. J Telemed Telecare.

[10] Baker LC, Johnson SJ, Macaulay D, Birnbaum H (2011). Integrated telehealth and care management program for Medicare beneficiaries with chronic disease linked to savings. Health Aff (Millwood).

[11] Kalwani NM, Johnson AN, Parameswaran V, Dash R, Rodriguez F (2021). Initial outcomes of CardioClick, a telehealth program for preventive cardiac care: observational study. JMIR Cardio.

[12] Greenhalgh J, Gooding K, Gibbons E, Dalkin S, Wright J, Valderas J, Black N (2018). How do Patient Reported Outcome Measures (PROMs) support clinician-patient communication and patient care? A realist synthesis. J Patient Rep Outcomes.

[13] Campbell R, Ju A, King MT, Rutherford C (2022). Perceived benefits and limitations of using patient-reported outcome measures in clinical practice with individual patients: a systematic review of qualitative studies. Qual Life Res.

[14] Wintner LM, Giesinger JM, Schauer-Maurer G, Holzner B, Hayat M (2011). Electronic Patient-Reported Outcome Monitoring (ePROM) in brain tumour patients. Tumors of the Central Nervous System, Volume 5.

[15] Meirte J, Hellemans N, Anthonissen M, Denteneer L, Maertens K, Moortgat P, Van Daele U (2020). Benefits and disadvantages of electronic patient-reported outcome measures: systematic review. JMIR Perioper Med.

[16] Cella D, Riley W, Stone A, Rothrock N, Reeve B, Yount S, Amtmann D, Bode R, Buysse D, Choi S, Cook K, Devellis R, DeWalt D, Fries JF, Gershon R, Hahn EA, Lai JS, Pilkonis P, Revicki D, Rose M, Weinfurt K, Hays R (2010). The Patient-Reported Outcomes Measurement Information System (PROMIS) developed and tested its first wave of adult self-reported health outcome item banks: 2005-2008. J Clin Epidemiol.

[17] DeWalt DA, Rothrock N, Yount S, Stone AA, PROMIS Cooperative Group (2007). Evaluation of item candidates: the PROMIS qualitative item review. Med Care.

[18] LoMartire R, Äng BO, Gerdle B, Vixner L (2020). Psychometric properties of Short Form-36 Health Survey, EuroQol 5-dimensions, and Hospital Anxiety and Depression Scale in patients with chronic pain. Pain.

[19] Cordier R, Brown T, Clemson L, Byles J (2018). Evaluating the longitudinal item and category stability of the SF-36 full and summary scales using Rasch analysis. Biomed Res Int.

[20] Hyman MA (2020). MSQ—Medical Symptom/Toxicity Questionnaire. DrHyman.com.

[21] Williams H, Steinberg S, Berzin R (2021). The development of a digital patient-reported outcome measurement for adults with chronic disease (the Parsley Symptom Index): prospective cohort study. JMIR Form Res.

[22] Williams H, Steinberg S, Leon K, O'Shea C, Berzin R, Hagg H (2022). Validity of the Parsley Symptom Index-an electronic patient-reported outcomes measure designed for telehealth: prospective cohort study. JMIR Form Res.

[23] Ramspek CL, Jager KJ, Dekker FW, Zoccali C, van Diepen M (2021). External validation of prognostic models: what, why, how, when and where?. Clin Kidney J.

[24] (2009). Patient-reported outcome measures: use in medical product development to support labeling claims. US Food and Drug Administration.

[25] Cook KF, Jensen SE, Schalet BD, Beaumont JL, Amtmann D, Czajkowski S, Dewalt DA, Fries JF, Pilkonis PA, Reeve BB, Stone AA, Weinfurt KP, Cella D (2016). PROMIS measures of pain, fatigue, negative affect, physical function, and social function demonstrated clinical validity across a range of chronic conditions. J Clin Epidemiol.

[26] Merriwether EN, Rakel BA, Zimmerman MB, Dailey DL, Vance CG, Darghosian L, Golchha M, Geasland KM, Chimenti R, Crofford LJ, Sluka KA (2017). Reliability and construct validity of the Patient-Reported Outcomes Measurement Information System (PROMIS) instruments in women with fibromyalgia. Pain Med.

[27] Howell CR, Gross HE, Reeve BB, DeWalt DA, Huang IC (2016). Known-groups validity of the Patient-Reported Outcomes Measurement Information System (PROMIS(®)) in adolescents and young adults with special healthcare needs. Qual Life Res.

[28] Fox GW, Rodriguez S, Rivera-Reyes L, Loo G, Hazan A, Hwang U (2020). PROMIS physical function 10-item short form for older adults in an emergency setting. J Gerontol A Biol Sci Med Sci.

[29] Lam KH, Kwa VIH (2018). Validity of the PROMIS-10 global health assessed by telephone and on paper in minor stroke and transient ischaemic attack in the Netherlands. BMJ Open.

[30] Pak SS, Miller MJ, Cheuy VA (2021). Use of the PROMIS-10 global health in patients with chronic low back pain in outpatient physical therapy: a retrospective cohort study. J Patient Rep Outcomes.

[31] Kahan JB, Kassam HF, Nicholson AD, Saad MA, Kovacevic D (2019). Performance of PROMIS Global-10 to legacy instruments in patients with lateral epicondylitis. Arthroscopy.

[32] Python Version 3.10.

[33] Hays RD, Schalet BD, Spritzer KL, Cella D (2017). Two-item PROMIS® global physical and mental health scales. J Patient Rep Outcomes.

[34] Northwestern University PROMIS®. HealthMeasures.

[35] Eysenbach G (2004). Improving the quality of web surveys: the Checklist for Reporting Results of Internet E-Surveys (CHERRIES). J Med Internet Res.

[36] Williams H, Steinberg S, Vingum R, Leon K, Céspedes E, Berzin R, Hagg H (2023). Parsley Health: feasibility and acceptability of a large-scale holistic telehealth program for chronic disease care. Front Digit Health.

