# Supplementary material for: Predictive Criterion Validity of the Parsley Symptom Index Against the Patient-Reported Outcomes Measurement Information System-10 in a Chronic Disease Cohort: Retrospective Cohort Study
Source: JMIR Form Res. 2024 Feb 16;8:e53316. doi: 10.2196/53316 (PMC10907938; doi:10.2196/53316)
Supplement: Multimedia Appendix 1 [file formative_v8i1e53316_app1.docx]

Appendix Item 1: PSI T-score Conversion Methodology and Table.

In Appendix 1 we present the methodology used to derive the T-score conversion table for the Parsley Symptom Index (PSI), from an independent dataset of customers from Parsley Health. While the main body of this manuscript focuses on a comparative analysis between PSI and PROMIS, this section provides a reference framework for the generation of the PSI T-score table. It is important for readers to recognize that the data from Parsley Health may exhibit unique characteristics and potential selection biases not evident in the clinical populations discussed in the main text of this manuscript, or generally in other clinical samples. The purpose of this appendix is to offer a detailed context for the PSI T-scores to ensure transparency, and to support its application within the scope of this manuscript.

The T-score table for the Parsley Symptom Index (PSI) was derived from 122,591 PSI assessments among 29,353 individuals from Parsley Health. Reverse coding was applied to align with Patient-Reported Outcomes Measurement Information System (PROMIS) standards, thereby facilitating a consistent interpretation across various patient-reported outcome measures where higher scores indicate healthier individuals.

| Original Value | Reverse Coded Value |
| --- | --- |
| 0 | 10 |
| 1 | 9 |
| 2 | 8 |
| 3 | 7 |
| 4 | 6 |
| 5 | 5 |
| 6 | 4 |
| 7 | 3 |
| 8 | 2 |
| 9 | 1 |
| 10 | 0 |

The post-recoding PSI scores for this sample had a mean of 439 (SD=37), with a minimum and maximum value range of 138 to 500. The distribution exhibited a kurtosis of 2.34, suggesting a sharper peak than a normal distribution, and a skewness of -1.27, indicating a moderate leftward asymmetry. These statistics underscore the non-normality of the PSI score distribution, justifying the need for a tailored approach to T-score transformation.

Traditional T-score transformations typically assume a normal distribution of scores, and may not adequately represent distributions with characteristics such as skewness, kurtosis, or outliers, which are observed in the PSI scores. Recognizing the non-normal distribution of PSI scores, we employed quartiles to partition the data into segments containing equal data proportions (code provided below). This quartile-based approach divides the distribution into four equal parts, each mapped to a distinct T-score range, to reflect the distribution's central tendency and variability more accurately than mean and standard deviation measures.

This segmentation ensures that T-scores not only represent the relative position of scores within the PSI population but also correspond to clinically relevant score ranges:

- First Quartile (0-25%): Mapping to T-scores from 20 to 35 for PSI scores below 420.
- Second Quartile (25-50%): Mapping to T-scores from 35 to 45 for PSI scores between 420 and 447.
- Third Quartile (50-75%): Mapping to T-scores from 45 to 55 for PSI scores between 447 and 467.
- Fourth Quartile (75-100%): Mapping to T-scores from 55 to 70 for PSI scores above 467.

It is important to note that this approach adopts linear interpolation within each quartile to map raw scores to T-scores, creating a piecewise linear relationship between raw scores and T-scores. This method is simpler and more practical than non-linear transformations, yet it provides a tailored fit to the PSI score distribution.

To enhance the clinical and research utility of our T-scores, we computed the standard error of measurement, indicating precision across the score range. Our final conversion table below details PSI scores and corresponding T-scores with standard errors and offers a pragmatic and precise tool for healthcare professionals and researchers to use in the future.

| Reverse Coded PSI Total Score | T-Score Conversion |
| --- | --- |
| 0 | 20 |
| 1 | 20.0357143 |
| 2 | 20.0714286 |
| 3 | 20.1071429 |
| 4 | 20.1428571 |
| 5 | 20.1785714 |
| 6 | 20.2142857 |
| 7 | 20.25 |
| 8 | 20.2857143 |
| 9 | 20.3214286 |
| 10 | 20.3571429 |
| 11 | 20.3928571 |
| 12 | 20.4285714 |
| 13 | 20.4642857 |
| 14 | 20.5 |
| 15 | 20.5357143 |
| 16 | 20.5714286 |
| 17 | 20.6071429 |
| 18 | 20.6428571 |
| 19 | 20.6785714 |
| 20 | 20.7142857 |
| 21 | 20.75 |
| 22 | 20.7857143 |
| 23 | 20.8214286 |
| 24 | 20.8571429 |
| 25 | 20.8928571 |
| 26 | 20.9285714 |
| 27 | 20.9642857 |
| 28 | 21 |
| 29 | 21.0357143 |
| 30 | 21.0714286 |
| 31 | 21.1071429 |
| 32 | 21.1428571 |
| 33 | 21.1785714 |
| 34 | 21.2142857 |
| 35 | 21.25 |
| 36 | 21.2857143 |
| 37 | 21.3214286 |
| 38 | 21.3571429 |
| 39 | 21.3928571 |
| 40 | 21.4285714 |
| 41 | 21.4642857 |
| 42 | 21.5 |
| 43 | 21.5357143 |
| 44 | 21.5714286 |
| 45 | 21.6071429 |
| 46 | 21.6428571 |
| 47 | 21.6785714 |
| 48 | 21.7142857 |
| 49 | 21.75 |
| 50 | 21.7857143 |
| 51 | 21.8214286 |
| 52 | 21.8571429 |
| 53 | 21.8928571 |
| 54 | 21.9285714 |
| 55 | 21.9642857 |
| 56 | 22 |
| 57 | 22.0357143 |
| 58 | 22.0714286 |
| 59 | 22.1071429 |
| 60 | 22.1428571 |
| 61 | 22.1785714 |
| 62 | 22.2142857 |
| 63 | 22.25 |
| 64 | 22.2857143 |
| 65 | 22.3214286 |
| 66 | 22.3571429 |
| 67 | 22.3928571 |
| 68 | 22.4285714 |
| 69 | 22.4642857 |
| 70 | 22.5 |
| 71 | 22.5357143 |
| 72 | 22.5714286 |
| 73 | 22.6071429 |
| 74 | 22.6428571 |
| 75 | 22.6785714 |
| 76 | 22.7142857 |
| 77 | 22.75 |
| 78 | 22.7857143 |
| 79 | 22.8214286 |
| 80 | 22.8571429 |
| 81 | 22.8928571 |
| 82 | 22.9285714 |
| 83 | 22.9642857 |
| 84 | 23 |
| 85 | 23.0357143 |
| 86 | 23.0714286 |
| 87 | 23.1071429 |
| 88 | 23.1428571 |
| 89 | 23.1785714 |
| 90 | 23.2142857 |
| 91 | 23.25 |
| 92 | 23.2857143 |
| 93 | 23.3214286 |
| 94 | 23.3571429 |
| 95 | 23.3928571 |
| 96 | 23.4285714 |
| 97 | 23.4642857 |
| 98 | 23.5 |
| 99 | 23.5357143 |
| 100 | 23.5714286 |
| 101 | 23.6071429 |
| 102 | 23.6428571 |
| 103 | 23.6785714 |
| 104 | 23.7142857 |
| 105 | 23.75 |
| 106 | 23.7857143 |
| 107 | 23.8214286 |
| 108 | 23.8571429 |
| 109 | 23.8928571 |
| 110 | 23.9285714 |
| 111 | 23.9642857 |
| 112 | 24 |
| 113 | 24.0357143 |
| 114 | 24.0714286 |
| 115 | 24.1071429 |
| 116 | 24.1428571 |
| 117 | 24.1785714 |
| 118 | 24.2142857 |
| 119 | 24.25 |
| 120 | 24.2857143 |
| 121 | 24.3214286 |
| 122 | 24.3571429 |
| 123 | 24.3928571 |
| 124 | 24.4285714 |
| 125 | 24.4642857 |
| 126 | 24.5 |
| 127 | 24.5357143 |
| 128 | 24.5714286 |
| 129 | 24.6071429 |
| 130 | 24.6428571 |
| 131 | 24.6785714 |
| 132 | 24.7142857 |
| 133 | 24.75 |
| 134 | 24.7857143 |
| 135 | 24.8214286 |
| 136 | 24.8571429 |
| 137 | 24.8928571 |
| 138 | 24.9285714 |
| 139 | 24.9642857 |
| 140 | 25 |
| 141 | 25.0357143 |
| 142 | 25.0714286 |
| 143 | 25.1071429 |
| 144 | 25.1428571 |
| 145 | 25.1785714 |
| 146 | 25.2142857 |
| 147 | 25.25 |
| 148 | 25.2857143 |
| 149 | 25.3214286 |
| 150 | 25.3571429 |
| 151 | 25.3928571 |
| 152 | 25.4285714 |
| 153 | 25.4642857 |
| 154 | 25.5 |
| 155 | 25.5357143 |
| 156 | 25.5714286 |
| 157 | 25.6071429 |
| 158 | 25.6428571 |
| 159 | 25.6785714 |
| 160 | 25.7142857 |
| 161 | 25.75 |
| 162 | 25.7857143 |
| 163 | 25.8214286 |
| 164 | 25.8571429 |
| 165 | 25.8928571 |
| 166 | 25.9285714 |
| 167 | 25.9642857 |
| 168 | 26 |
| 169 | 26.0357143 |
| 170 | 26.0714286 |
| 171 | 26.1071429 |
| 172 | 26.1428571 |
| 173 | 26.1785714 |
| 174 | 26.2142857 |
| 175 | 26.25 |
| 176 | 26.2857143 |
| 177 | 26.3214286 |
| 178 | 26.3571429 |
| 179 | 26.3928571 |
| 180 | 26.4285714 |
| 181 | 26.4642857 |
| 182 | 26.5 |
| 183 | 26.5357143 |
| 184 | 26.5714286 |
| 185 | 26.6071429 |
| 186 | 26.6428571 |
| 187 | 26.6785714 |
| 188 | 26.7142857 |
| 189 | 26.75 |
| 190 | 26.7857143 |
| 191 | 26.8214286 |
| 192 | 26.8571429 |
| 193 | 26.8928571 |
| 194 | 26.9285714 |
| 195 | 26.9642857 |
| 196 | 27 |
| 197 | 27.0357143 |
| 198 | 27.0714286 |
| 199 | 27.1071429 |
| 200 | 27.1428571 |
| 201 | 27.1785714 |
| 202 | 27.2142857 |
| 203 | 27.25 |
| 204 | 27.2857143 |
| 205 | 27.3214286 |
| 206 | 27.3571429 |
| 207 | 27.3928571 |
| 208 | 27.4285714 |
| 209 | 27.4642857 |
| 210 | 27.5 |
| 211 | 27.5357143 |
| 212 | 27.5714286 |
| 213 | 27.6071429 |
| 214 | 27.6428571 |
| 215 | 27.6785714 |
| 216 | 27.7142857 |
| 217 | 27.75 |
| 218 | 27.7857143 |
| 219 | 27.8214286 |
| 220 | 27.8571429 |
| 221 | 27.8928571 |
| 222 | 27.9285714 |
| 223 | 27.9642857 |
| 224 | 28 |
| 225 | 28.0357143 |
| 226 | 28.0714286 |
| 227 | 28.1071429 |
| 228 | 28.1428571 |
| 229 | 28.1785714 |
| 230 | 28.2142857 |
| 231 | 28.25 |
| 232 | 28.2857143 |
| 233 | 28.3214286 |
| 234 | 28.3571429 |
| 235 | 28.3928571 |
| 236 | 28.4285714 |
| 237 | 28.4642857 |
| 238 | 28.5 |
| 239 | 28.5357143 |
| 240 | 28.5714286 |
| 241 | 28.6071429 |
| 242 | 28.6428571 |
| 243 | 28.6785714 |
| 244 | 28.7142857 |
| 245 | 28.75 |
| 246 | 28.7857143 |
| 247 | 28.8214286 |
| 248 | 28.8571429 |
| 249 | 28.8928571 |
| 250 | 28.9285714 |
| 251 | 28.9642857 |
| 252 | 29 |
| 253 | 29.0357143 |
| 254 | 29.0714286 |
| 255 | 29.1071429 |
| 256 | 29.1428571 |
| 257 | 29.1785714 |
| 258 | 29.2142857 |
| 259 | 29.25 |
| 260 | 29.2857143 |
| 261 | 29.3214286 |
| 262 | 29.3571429 |
| 263 | 29.3928571 |
| 264 | 29.4285714 |
| 265 | 29.4642857 |
| 266 | 29.5 |
| 267 | 29.5357143 |
| 268 | 29.5714286 |
| 269 | 29.6071429 |
| 270 | 29.6428571 |
| 271 | 29.6785714 |
| 272 | 29.7142857 |
| 273 | 29.75 |
| 274 | 29.7857143 |
| 275 | 29.8214286 |
| 276 | 29.8571429 |
| 277 | 29.8928571 |
| 278 | 29.9285714 |
| 279 | 29.9642857 |
| 280 | 30 |
| 281 | 30.0357143 |
| 282 | 30.0714286 |
| 283 | 30.1071429 |
| 284 | 30.1428571 |
| 285 | 30.1785714 |
| 286 | 30.2142857 |
| 287 | 30.25 |
| 288 | 30.2857143 |
| 289 | 30.3214286 |
| 290 | 30.3571429 |
| 291 | 30.3928571 |
| 292 | 30.4285714 |
| 293 | 30.4642857 |
| 294 | 30.5 |
| 295 | 30.5357143 |
| 296 | 30.5714286 |
| 297 | 30.6071429 |
| 298 | 30.6428571 |
| 299 | 30.6785714 |
| 300 | 30.7142857 |
| 301 | 30.75 |
| 302 | 30.7857143 |
| 303 | 30.8214286 |
| 304 | 30.8571429 |
| 305 | 30.8928571 |
| 306 | 30.9285714 |
| 307 | 30.9642857 |
| 308 | 31 |
| 309 | 31.0357143 |
| 310 | 31.0714286 |
| 311 | 31.1071429 |
| 312 | 31.1428571 |
| 313 | 31.1785714 |
| 314 | 31.2142857 |
| 315 | 31.25 |
| 316 | 31.2857143 |
| 317 | 31.3214286 |
| 318 | 31.3571429 |
| 319 | 31.3928571 |
| 320 | 31.4285714 |
| 321 | 31.4642857 |
| 322 | 31.5 |
| 323 | 31.5357143 |
| 324 | 31.5714286 |
| 325 | 31.6071429 |
| 326 | 31.6428571 |
| 327 | 31.6785714 |
| 328 | 31.7142857 |
| 329 | 31.75 |
| 330 | 31.7857143 |
| 331 | 31.8214286 |
| 332 | 31.8571429 |
| 333 | 31.8928571 |
| 334 | 31.9285714 |
| 335 | 31.9642857 |
| 336 | 32 |
| 337 | 32.0357143 |
| 338 | 32.0714286 |
| 339 | 32.1071429 |
| 340 | 32.1428571 |
| 341 | 32.1785714 |
| 342 | 32.2142857 |
| 343 | 32.25 |
| 344 | 32.2857143 |
| 345 | 32.3214286 |
| 346 | 32.3571429 |
| 347 | 32.3928571 |
| 348 | 32.4285714 |
| 349 | 32.4642857 |
| 350 | 32.5 |
| 351 | 32.5357143 |
| 352 | 32.5714286 |
| 353 | 32.6071429 |
| 354 | 32.6428571 |
| 355 | 32.6785714 |
| 356 | 32.7142857 |
| 357 | 32.75 |
| 358 | 32.7857143 |
| 359 | 32.8214286 |
| 360 | 32.8571429 |
| 361 | 32.8928571 |
| 362 | 32.9285714 |
| 363 | 32.9642857 |
| 364 | 33 |
| 365 | 33.0357143 |
| 366 | 33.0714286 |
| 367 | 33.1071429 |
| 368 | 33.1428571 |
| 369 | 33.1785714 |
| 370 | 33.2142857 |
| 371 | 33.25 |
| 372 | 33.2857143 |
| 373 | 33.3214286 |
| 374 | 33.3571429 |
| 375 | 33.3928571 |
| 376 | 33.4285714 |
| 377 | 33.4642857 |
| 378 | 33.5 |
| 379 | 33.5357143 |
| 380 | 33.5714286 |
| 381 | 33.6071429 |
| 382 | 33.6428571 |
| 383 | 33.6785714 |
| 384 | 33.7142857 |
| 385 | 33.75 |
| 386 | 33.7857143 |
| 387 | 33.8214286 |
| 388 | 33.8571429 |
| 389 | 33.8928571 |
| 390 | 33.9285714 |
| 391 | 33.9642857 |
| 392 | 34 |
| 393 | 34.0357143 |
| 394 | 34.0714286 |
| 395 | 34.1071429 |
| 396 | 34.1428571 |
| 397 | 34.1785714 |
| 398 | 34.2142857 |
| 399 | 34.25 |
| 400 | 34.2857143 |
| 401 | 34.3214286 |
| 402 | 34.3571429 |
| 403 | 34.3928571 |
| 404 | 34.4285714 |
| 405 | 34.4642857 |
| 406 | 34.5 |
| 407 | 34.5357143 |
| 408 | 34.5714286 |
| 409 | 34.6071429 |
| 410 | 34.6428571 |
| 411 | 34.6785714 |
| 412 | 34.7142857 |
| 413 | 34.75 |
| 414 | 34.7857143 |
| 415 | 34.8214286 |
| 416 | 34.8571429 |
| 417 | 34.8928571 |
| 418 | 34.9285714 |
| 419 | 34.9642857 |
| 420 | 35 |
| 421 | 35.3703704 |
| 422 | 35.7407407 |
| 423 | 36.1111111 |
| 424 | 36.4814815 |
| 425 | 36.8518519 |
| 426 | 37.2222222 |
| 427 | 37.5925926 |
| 428 | 37.962963 |
| 429 | 38.3333333 |
| 430 | 38.7037037 |
| 431 | 39.0740741 |
| 432 | 39.4444444 |
| 433 | 39.8148148 |
| 434 | 40.1851852 |
| 435 | 40.5555556 |
| 436 | 40.9259259 |
| 437 | 41.2962963 |
| 438 | 41.6666667 |
| 439 | 42.037037 |
| 440 | 42.4074074 |
| 441 | 42.7777778 |
| 442 | 43.1481481 |
| 443 | 43.5185185 |
| 444 | 43.8888889 |
| 445 | 44.2592593 |
| 446 | 44.6296296 |
| 447 | 45 |
| 448 | 45.5 |
| 449 | 46 |
| 450 | 46.5 |
| 451 | 47 |
| 452 | 47.5 |
| 453 | 48 |
| 454 | 48.5 |
| 455 | 49 |
| 456 | 49.5 |
| 457 | 50 |
| 458 | 50.5 |
| 459 | 51 |
| 460 | 51.5 |
| 461 | 52 |
| 462 | 52.5 |
| 463 | 53 |
| 464 | 53.5 |
| 465 | 54 |
| 466 | 54.5 |
| 467 | 55 |
| 468 | 55.4545455 |
| 469 | 55.9090909 |
| 470 | 56.3636364 |
| 471 | 56.8181818 |
| 472 | 57.2727273 |
| 473 | 57.7272727 |
| 474 | 58.1818182 |
| 475 | 58.6363636 |
| 476 | 59.0909091 |
| 477 | 59.5454545 |
| 478 | 60 |
| 479 | 60.4545455 |
| 480 | 60.9090909 |
| 481 | 61.3636364 |
| 482 | 61.8181818 |
| 483 | 62.2727273 |
| 484 | 62.7272727 |
| 485 | 63.1818182 |
| 486 | 63.6363636 |
| 487 | 64.0909091 |
| 488 | 64.5454545 |
| 489 | 65 |
| 490 | 65.4545455 |
| 491 | 65.9090909 |
| 492 | 66.3636364 |
| 493 | 66.8181818 |
| 494 | 67.2727273 |
| 495 | 67.7272727 |
| 496 | 68.1818182 |
| 497 | 68.6363636 |
| 498 | 69.0909091 |
| 499 | 69.5454545 |
| 500 | 70 |

***Python Script for Computation:***

## load in data as df

df = pd.read_csv(data.csv')

## generated quartiles

q1 = df.psi_score_reversed.quantile(0.25)

q2 = df.psi_score_reversed.quantile(0.50)

q3 = df.psi_score_reversed.quantile(0.75)

def convert_to_custom_t_score(raw_score):

if raw_score < 420.0:

t_score = 20 + 15 * (raw_score / 420.0)

elif raw_score < 447.0:

t_score = 35 + 10 * ((raw_score - 420.0) / (447.0 - 420.0))

elif raw_score < 467.0:

t_score = 45 + 10 * ((raw_score - 447.0) / (467.0 - 447.0))

else:

t_score = 55 + 15 * ((raw_score - 467.0) / (500.0 - 467.0))

return t_score

### for generating standard error of measurement

def t_score_slope(raw_score):

if raw_score < 420.0:

slope = 15 / 420.0

elif raw_score < 447.0:

slope = 10 / (447.0 - 420.0)

elif raw_score < 467.0:

slope = 10 / (467.0 - 447.0)

else:

slope = 15 / (500.0 - 467.0)

return slope

# Create a DataFrame to hold the conversion table

conversion_table = pd.DataFrame({

'raw_score': range(501), # Raw scores from 0 to 500

})

conversion_table['t_score'] = conversion_table['raw_score'].apply(convert_to_custom_t_score)

## save to csv

conversion_table.to_csv('data/psi_conversion_table.csv', index=False)
