# Supplementary material for: Predictive Criterion Validity of the Parsley Symptom Index Against the Patient-Reported Outcomes Measurement Information System-10 in a Chronic Disease Cohort: Retrospective Cohort Study
Source: JMIR Form Res. 2024 Feb 16;8:e53316. doi: 10.2196/53316 (PMC10907938; doi:10.2196/53316)
Supplement: Multimedia Appendix 2 [file formative_v8i1e53316_app2.pdf]

Item Category	Checklist Item	Description	Explanation - for PSI Study	Page Number/Section
	Describe Survey Design	Design	Retrospective Cohort Study	Methods
**IRB (Institutional Review Board) approval and informed consent process**	IRB approval	Mention whether the study has been approved by an IRB.	IRB approved	Ethics
	Informed Consent	Describe the informed consent process. Where were the participants told the length of time of the survey, which data were stored and where and for how long, who the investigator was, and the purpose of the study?	Exempt	Ethics
	Data Protection	If any personal information was collected or stored, describe what mechanisms were used to protect unauthorized access.	refer to citation 21 for data protection methods.	references
Development and pre-testing	Development and Testing	State how the survey was developed, including whether the usability and technical functionality of the electronic questionnaire had been tested before fielding the questionnaire.	Survey development is described in Williams H, Steinberg S, Berzin R. The development of a digital patient-reported outcome measurement for adults with chronic disease (The Parsley Symptom Index): Prospective cohort study. JMIR Formative Research. 2021;5(6). doi: 10.2196/29122	references
**Recruitment process and description of the sample having access to the questionnaire**	Open Survey v closed survey	An “open survey” is a survey open for each visitor of a site, while a closed survey is only open to a sample which the investigator knows (password-protected survey).	closed survey	Methods
	contact mode	Indicate whether or not the initial contact with the potential participants was made on the Internet. (Investigators may also send out questionnaires by mail and allow for Web-based data entry.)	Participants were new clinic patients.	Methods
	administering the survey	How/where was the survey announced or advertised? Some examples are offline media (newspapers), or online (mailing lists – If yes, which ones?) or banner ads (Where were these banner ads posted and what did they look like?). It is important to know the wording of the announcement as it will heavily influence who chooses to participate. Ideally the survey announcement should be published as an appendix.	Not advertised - administered as part of the clinic intake process	Methods
Survey Administration	Web/E-mail	State the type of e-survey (eg, one posted on a Web site, or one sent out through e-mail). If it is an e-mail survey, were the responses entered manually into a database, or was there an automatic method for capturing responses?	Web	Methods

	Context	Describe the Web site (for mailing list/newsgroup) in which the survey was posted. What is the Web site about, who is visiting it, what are visitors normally looking for? Discuss to what degree the content of the Web site could pre-select the sample or influence the results. For example, a survey about vaccination on a anti-immunization Web site will have different results from a Web survey conducted on a government Web site	The site is the electronic patient portal	Methods
	Mandatory/voluntary	Was it a mandatory survey to be filled in by every visitor who wanted to enter the Web site, or was it a voluntary survey?	mandatory for first visit, requested and encouraged for follow ups	Methods
	incentives	Were any incentives offered (eg, monetary, prizes, or non-monetary incentives such as an offer to provide the survey results)?	no monetary incentives. Feedback of 1) doing survey 2) discussing survey with clinician 3) reviewing results over time with clinician (graph	Methods
	time/date	In what timeframe were the data collected?	August 30, 2017 and Jan 30, 2023	Methods
	randomization of items or questionnaires	To prevent biases items can be randomized or alternated.	order of PSI and PROMIS1-10 not randomized due to technical limitations of system. Retrospective cohort, so couldn't change randomization retrospectively.	
	adaptive questioning	Use adaptive questioning (certain items, or only conditionally displayed based on responses to other items) to reduce number and complexity of the questions.	yes	
	number of items	What was the number of questionnaire items per page? The number of items is an important factor for the completion rate.	Due to the design of MDHQ (the EHR used), all PROMIS items appeared on the same page. For the PSI, patients completed one item per page.	
	number of screens (pages)	Over how many pages was the questionnaire distributed? The number of items is an important factor for the completion rate.	48 - 93 depending on adaptive questions/conditional responses	
	completeness check	It is technically possible to do consistency or completeness checks before the questionnaire is submitted. Was this done, and if "yes", how (usually JAVAScript)? An alternative is to check for completeness after the questionnaire has been submitted (and highlight mandatory items). If this has been done, it should be reported. All items should provide a non-response option such as "not applicable" or "rather not say", and selection of one response option should be enforced.	Yes, for the PSI a dedicated team of software engineers (internal) performed pre go-live checks on data validation and collection	reference 21

	Review step	State whether respondents were able to review and change their answers (eg, through a Back button or a Review step which displays a summary of the responses and asks the respondents if they are correct).	Yes - Participants have the option of using the back button to change answers prior to submitting. Once submitted, patients are unable to change their answers.	
Response rates	Unique site visitor	If you provide view rates or participation rates, you need to define how you determined a unique visitor. There are different techniques available, based on IP addresses or cookies or both.	Survey completed through online portal that requires registration and login for repeated visits.	methods
	View rate (Ratio of unique survey visitors/unique site visitors)	Requires counting unique visitors to the first page of the survey, divided by the number of unique site visitors (not page views!). It is not unusual to have view rates of less than 0.1 % if the survey is voluntary.	N/A	
	Participation rate (Ratio of unique visitors who agreed to participate/unique first survey page visitors)	The number of people submitting the last questionnaire page, divided by the number of people who agreed to participate (or submitted the first survey page). This is only relevant if there is a separate “informed consent” page or if the survey goes over several pages. This is a measure for attrition. Note that “completion” can involve leaving questionnaire items blank. This is not a measure for how completely questionnaires were filled in. (If you need a measure for this, use the word “completeness rate”.)	Average completion rate was 3.2 (PSI) and 3.4 (PROMIS) over the study period.	Results
**Preventing multiple entries from the same individual**	Cookies used	Indicate whether cookies were used to assign a unique user identifier to each client computer. If so, mention the page on which the cookie was set and read, and how long the cookie was valid. Were duplicate entries avoided by preventing users access to the survey twice; or were duplicate database entries having the same user ID eliminated before analysis? In the latter case, which entries were kept for analysis (eg, the first entry or the most recent)?	N/A	
	IP check		N/A; multi-factor authetnication is required to login	
	Log file analysis		N/A	
	Registration		Yes, registering is required	Methods
**Analysis**	Handling of incomplete questionnaires	Were only completed questionnaires analyzed? Were questionnaires which terminated early (where, for example, users did not go through all questionnaire pages) also analyzed?	PSI and PROMIS-10 has to be complete to be included in sample population per inclusion criteria.	Methods

	Questionnaires submitted with an atypical timestamp	Some investigators may measure the time people needed to fill in a questionnaire and exclude questionnaires that were submitted too soon. Specify the timeframe that was used as a cut-off point, and describe how this point was determined.	No assesments were excluded based on time receipts.	Methods
	Statistical correction	Indicate whether any methods such as weighting of items or propensity scores have been used to adjust for the non-representative sample; if so, please describe the methods.	No item weight was used or adjustment for non-represenitive sample.	Methods
